# Behind the Curtain: The Nurse’s Voice in Assessment of Residents in the Emergency Department

**DOI:** 10.5811/westjem.2018.10.39821

**Published:** 2018-11-19

**Authors:** Ashley Palvic, Dana Liu, Kara Baker, Joseph House, Michael Byrd, Tina Martinek, Diana O’Leary, Sally A. Santen

**Affiliations:** * University of Michigan Medical School, Department of Emergency Medicine, Ann Arbor, Michigan; † Wake Forest School of Medicine, Department of Emergency Medicine, Winston-Salem, North Carolina; ‡ North Shore University Hospital, Department of Emergency Medicine, Manhasset, New York; § Virginia Commonwealth University School of Medicine, Department of Emergency Medicine, Richmond, Virginia

## Abstract

**Introduction:**

Feedback provides valuable input for improving physician performance. Conventionally, feedback is obtained from attending physicians; however, residents work in close contact with other members of the care team, especially nurses. Nurses may have more opportunity to directly observe trainees. In addition, they may value different behaviors and provide unique feedback. The objective of this study was to examine the nurse’s perspective of resident performance in the emergency department.

**Methods:**

This was a retrospective, mixed-methods study of nursing assessments of residents using a five-point scale from 1 (unsatisfactory) to 5 (outstanding) and providing comments. Analysis included descriptive statistics of the quantitative assessments and content analysis of the nursing comments by a group of attendings, residents, and nurses.

**Results:**

Nurses assessed residents as above expectation or outstanding, especially for the categories of “How would you rate this resident’s attitude?” (65%) and “Is this resident a team player?” (64%). Content analysis of the comments yielded nine themes including being kind, communication with nurses, being a team player, work ethic and efficiency, and respect for other team members. Of the comments made, 50% provided positive feedback, and the majority of comments (80%) were determined to be actionable.

**Conclusion:**

Our data indicate that nurses provide feedback on residents’ kindness, efficiency and communication. These two aspects of interacting in the healthcare setting may not be highlighted in conventional, attending provider feedback, yet they are clearly noted by the nurse’s voice.

## INTRODUCTION

As self-assessment can be flawed, feedback is a valuable input for physician performance improvement.^1^ Conventionally, feedback is obtained from residents’ attending physicians. However, physicians work in close contact with other members of the care team, most prominently the nursing staff. Nurses may have more opportunity to directly observe residents performing patient care, including aspects of patient care that attending physicians do not routinely observe. Additionally, as nurses approach patient care from a different perspective, they may observe and remark on different behaviors and attitudes of the residents with whom they interact.^2^

Recognizing the importance of performance input from a variety of sources,^3^ many of the Accreditation Council for Graduate Medical Education (ACGME) Milestones recommend multi-source feedback, which is further supported by a mandate from the Emergency Medicine (EM) Residency Review Committee. Nursing perspective is particularly relevant to several of the EM milestones.^4^ These include the following: 1) effective communication; 2) working effectively as part of a healthcare team; 3) professionalism; and 4) systems-based practice, including the ability to work in interprofessional teams to enhance patient safety and quality.

Previous studies in other specialties have found that nursing assessments of residents are reliable and may provide information that is different from that provided by attendings.^2^,^5^–^10^ One study showed that nursing assessments of residents mirror patient assessments, unlike attending, peer, or self-assessments.^7^ Another study demonstrated that nurses were able to assess the humanistic qualities of residents, such as respect and integrity.^6^ Nurses may be less lenient than attendings, although still correlated.^8^ Additionally, nursing assessments of interpersonal skills correlated better with faculty measures, whereas assessments of medical knowledge did not correlate as well.^11^ These studies demonstrate the unique and concordant assessment domains compared to standard faculty assessments.

The literature demonstrates that the nursing perspective is both valid and at times correlated with other forms of assessment. Previous studies have not explored the specific behaviors nurses may observe and upon which they may comment. All of the previous studies included an assessment form with quantitative data points, rather than narrative, qualitative data points. The objective of this study was to examine the nurse’s perspective of resident performance in the emergency department (ED) by a quantitative analysis of assessments and content analysis of narrative comments. This project will contribute to our understanding of nursing narrative feedback to residents.

## METHODS

This was a retrospective, cross-sectional, mixed-method study of nursing assessments submitted from July 2010 to October 2013. The setting was an academic, four-year EM program with over 50 residents. Participants were nurses who worked in the ED and completed resident assessments using an online instrument with quantitative and narrative components. The quantitative component asked nurses to score residents on eight items (Table 1), rating them from 1 (unsatisfactory) to 5 (outstanding). Additionally, nurses were invited to provide narrative comments. The assessments were completed online (Medhub^TM^) and were not mandatory. Resident leadership periodically spoke at nursing staff meetings to encourage completion of the assessments. The residents receive these de-identified, aggregated, nurse assessments at each mandatory semi-annual review. The institutional review board determined the study exempt.

**Population Health Research Capsule**

What do we already know about this issue?

*Multi-source feedback is important because self- assessment is flawed. Specifically nursing feedback can be meaningful to guide resident behavior*.

What was the research question?

*What is the nursing perspective on emergency medicine (EM) resident behaviors? This study examined nursing assessments of EM residents*.

What was the major finding of the study?

*Nurses provided feedback and valued EM residents when they were kind, efficient, team players and communicated well*.

How does this improve population health?

*Nurses can provide meaningful feedback to residents to help improve patient care and teamwork*.

Quantitative analysis included descriptive statistics of 1,506 assessment forms to support the findings of the qualitative analysis. The narrative comments were de-identified and analyzed using content analysis.^12^–^16^ The analysis was informed by the literature on multisource feedback and by the expertise of the coding group (two nurses, four residents, and two faculty, including a qualitative expert). We started with team immersion review of the data. From this, we developed an initial set of codes. Given the different perspectives, coding was then done iteratively as a group over multiple sessions using the constant comparative method of analysis and grouping of data chunks. When the team disagreed on how a comment should be coded, this was resolved through dialogue. We recorded and refined emergent themes. Saturation was achieved, as no new themes emerged after the first 150 comments (assessment questionnaires) were coded. We coded an additional 60 for a total of 210 to ensure no new themes. Themes are presented using the nurse’s written voice.

## RESULTS

### Qualitative Data

For all comments two themes were determined. The first was whether the comment provided feedback that was positive or negative. Based on content analysis, 50% of comments were positive, 50% were negative, and 10% were coded as both. The second theme was whether feedback from the comment was actionable or not. Actionable comments were those that were specific enough that the resident could conceivably choose to change behavior to act upon the comment. The majority of comments (80%) were determined to be actionable. An example of actionable is “This MD might improve by being better aware of the patient care that is completed by the registered nurse (RN) and the timeline it takes to accomplish some tasks.”

We identified nine additional themes (Table 2). The most common themes were *nice/kind*, *communication with nurses*, and *work ethic/efficiency* (Table 1). Nurses described the residents both in positive and negative behaviors for each of these themes. The following section will describe the most common themes with direct quotations to demonstrate and clarify the theme.

Nurses frequently commented on *communication with nurses*. This included updating and informing nurses on the plan of care, new orders or tasks, and being responsive to pages. For example, one positive comment mentioned, “He works great with the nurses and keeps them informed of the treatment plan.” On the other hand, nurses noticed when this did not occur: “Does not initiate conversations regarding patient care/updates with staff.” Multiple nurses commented specifically on residents not responding to pages promptly. “He rarely responds to pages. It is very difficult to get in touch with him regarding questions and requests. I usually have to give up and go find him, which can be frustrating.”

Another common theme was the *work ethic and efficiency* of the resident. Nurses frequently commented on whether residents were able to pick up, evaluate, and disposition patients in a timely manner, as well as the ability to multitask, prioritize, and balance patient load: “Very unorganized. Takes long time to dispo[sition] patients.” Many nurses commented on whether residents completed orders in a timely and efficient fashion, specifically whether they placed all necessary orders at one time or staggered them. “Gives verbal orders but doesn’t follow through with written orders.” “MD should order appropriately per patient condition and relay to RN. There were many instances with Dr. [ ] where RN had to request imaging orders on a critical patient because she failed to write them – putting patient at risk.”

The third theme was *nice/kind*. Nurses referred to these residents as being generally enjoyable to work with and having qualities such as being approachable, friendly and taking time to address questions and concerns of ED staff. The opposite was being rude, brusque, and unapproachable. Some examples include “Very approachable and great to work with,” and “She is not very pleasant to work with […] and also is very short with the nursing staff.”

*Resident judgment* included comments on residents’ knowledge base and decision-making capabilities as well as on their procedural skills. Nurses commented on whether residents had the judgment to recognize critical patients and give appropriate guidance to nursing. “I had a patient who was very hypotensive and hypoxic, and he left me with the patient to go to another patient in resus[citation]…he did not give any direction or send in another physician.” The nurses also made comments on residents’ skills such as, “Awesome pt (patient) positioning when it comes to suturing!” and “Does not know how to administer eye meds.”

Nurses are often present as residents *communicate with patients and families*. The nurses noted whether residents had good bedside manner, developed rapport with patients, and updated patients on the plan of care. In a positive example, a nurse commented, “Dr. [ ] has the ability to communicate with his patient and family in a way that informs, encourages, and teaches [… ] and asks if there is anything more that the patient or family needs.” On the other hand, nurses noticed when residents were not communicating with their patients, such as, “I have been put in situations where my patients have wanted to leave because she had not seen them in hours after ordering exams for them.” As nurses often go through a resident’s discharge instructions with patients, they were able to comment on those as well.

Nurses also commented on whether the resident was a *team player*. This included how the resident worked with all the staff and whether he or she did tasks outside of the usual job of the doctor to help patient care. “He is a fantastic MD - he helped me start a difficult IV - he was helpful and respectful of my time.” Another positive example included, “He is one of the few MDs who will help a [patient] walk to the restroom or get them a blanket. He genuinely seems to be a team player, and I appreciate the help he has given me in my patient care.” On the other hand, they note when residents are not working well with other members of the team, as evidenced by “does not work with other staff well, just tells them what to do in a strict ‘I am better then you’ attitude,” and another, “Would like the resident to be more of a team player and supportive of the nurses with combative patients.”

Nurses note *leadership and confidence*. Specific attributes that the nurses commented upon included decisiveness in voicing orders and plan of care, staying calm in difficult situations, and answering questions with certainty. On the other hand, a lack of confidence included those who were anxious, appeared stressed and unsure of themselves, and were not specific in voicing orders and plan of care. This category also included leadership, and when this was commented upon, it was often in the context of running resuscitations. “Dr. [ ] continues to appear/act in a passive manner while working in the resus[citation] bays. He does not direct well or take on a leadership role during critical times.”

There were multiple comments on the manner in which the residents communicated. These were categorized as *respect for other team members*. For example, “Dr. [ ] is condescending to staff and rolls her eyes constantly,” and “demonstrates too much arrogance. Does not appear like he wants to listen to nursing staff, not important [sic].” Many of these comments were closely tied to comments about the resident being a team player.

A final category included comments about acknowledging *nursing clinical judgment*. The nurses wanted residents to be open to their suggestions or opinions on patient care and to listen when they expressed concerns. Positive assessments included statements such as “Seems to respect the information that the RN brings to the patient,” and “Able to accept questioning of orders from nursing staff…listens to suggestions when offered.” In contrast, nurses were aware of and commented about residents who did not acknowledge nursing concerns. “Would like it if he would take the nurse’s views, observation into consideration instead of acting solely.” This category also included comments about recognizing the nurses’ patient load, time constraints and having a good understanding of what nurses in the ED are supposed to do.

### Quantitative Data

The quantitative results are found in Table 2. Generally, nurses scored physicians above expectation, especially for the categories of “How would you rate this resident’s attitude?” and “Is this resident a team player?” Residents were scored lowest on “How would you rate this resident’s clinical efficiency and ability to maintain patient flow?”

## DISCUSSION

The nurse’s voice in assessment of residents provided unique perspectives and feedback for residents. Their comments suggest that nurses note good communication and the relationship between nurses and doctors (kindness). The advantage to the qualitative analysis of the comments is that they provide a deeper understanding of what nurses observe in the behavior of residents. For example, while we may feel that we understand what “efficiency” means, the specific comments help to enrich our understanding (e.g., putting in all orders at one time so that the nurse does not have to duplicate work by redrawing blood or contacting the laboratory).

The quantitative numbers were primarily positive; however, the narrative comments included a number of negative comments. It is possible that for the survey the nurses may have been providing a socially desirable response by scoring the residents highly. While there is some overlap between the narrative comments and the quantitative questions, nurses also provided additional information to the residents through their voice. Further, there is likely the recognition in the comments that there are still areas of improvement for the residents.

When providing feedback, Tekian et al. noted the importance of linking feedback to an action plan.^17^ We found that the majority of nursing assessments comments are actionable and can be as simple as entering lab orders at the same time so that nurses do not have to redraw blood, to more thoughtful behaviors such as getting a patient a warm blanket when the resident recognizes that the nurse is currently busy with multiple pressing tasks. There was a consensus among nursing comments in terms of the specific behaviors directly observed in the clinical setting. The most salient qualities that we found nurses to note were the following: 1) placing orders promptly and at the same time; 2) communicating the plan of care directly with nurses; 3) communicating results and plan of care with patients; and 4) responding to nursing concerns and pages in a timely manner.

Additionally, nurses may also identify patterns of physician behavior that could potentially be detrimental to residents’ professional advancement (e.g., speaking to nurses and patients in a condescending tone). The residents may not be aware that this perception or their behavior is negatively affecting others. By highlighting what is important – attitude, teamwork and efficiency – nursing comments could provide stimulus through which residents can inform their own self-assessment and make positive changes. Bringing nurses into the conversation helps physician providers understand domains of performance of which they may not be aware and promotes an interdisciplinary approach to the assessment of residents in the clinical setting that may lead residents to improved self-assessment and team dynamics.

The Joint Commission’s report on sentinel events demonstrated that in the majority of events, issues with communication were one of the major root causes.^18^ In a culture of safety, attention is focused on effective teamwork and communication between healthcare providers. Therefore, as nurses provide actionable feedback through their comments provided to residents, they are instructing the residents how to become better members of the team. Residents who can incorporate this feedback may have improved interactions with the team and be able to provide improved patient care. The use of nursing feedback in resident assessment by residency programs also indicates the importance of our nursing partners and their role in patient care and the team.

Conventionally, resident feedback comes predominantly from the attending physicians. They are appropriately situated to assess a resident’s procedural skills and medical knowledge; however, the resident’s learning environment is broader than the attending-resident interaction. To be an effective physician, residents must also display characteristics such as interpersonal and communication skills, professionalism, and systems-based practice.^15^ The interactions in which residents display these characteristics may occur more often with other members of the care team as well as with the patients—interactions that attending physicians do not frequently observe. Nursing assessments add color, depth, and context to resident assessments and, when used in conjunction with conventional attending provider feedback, may provide a more holistic picture of a resident’s ability to provide effective patient care. Further studies need to compare the comments and scores of the nurses, faculty, and peers for each resident. In addition, it will be important to examine the design of assessments specifically for the purpose of providing feedback.^17^ While this paper examined nursing feedback for residents, it is also important to include patient feedback in the multisource feedback for residents.

Future studies should examine if residents’ reviews of actionable nursing assessments influence a change in their behavior. From what we know about poor self-assessment, the nursing comments should be part of informed self-assessment.^19^,^20^ If residents were to identify nurses as a respected, “trusted source,” nurses could then conceivably help coach those residents with problematic behavior. For example, when a resident does not understand how s/he might be perceived as arrogant, the nursing coach could help provide specific examples and better approaches.

## LIMITATIONS

There were several limitations associated with this study. First, the nursing survey contained the eight questions on specific characteristics of physicians before the comment, free-text portion. These specific questions may have influenced the free-text responses. Because we do not have a response rate for nurses completing evaluations there may be some bias. In addition, there may be some social desirability response in the textual comments. Second, this was a single site, which may constrain generalizability. This study did not examine the changes over time for the responses, as the nursing assessments of residents started over a decade ago. Finally, this study is an initial step toward understanding nurses’ feedback to residents; however, there are limitations implicit in our qualitative methods. Qualitative studies are not intended to test inferences about causation or associations.

## CONCLUSION

Nurse-physician relationships form the basis of effective interprofessional practice and patient care. Nurses’ comments suggest that they remark on communication and the relationship between nurses and doctors as well as teamwork and efficiency. Nurses’ assessments can provide feedback and direction for resident professional development.

## Figures and Tables

**Table 1:** Nurses’ quantitative assessments of residents.

	Outstanding/ above expectation (5 and 4)	At expectation (3)	Below expectation/ unsatisfactory (1 and 2)	Mean (SD)
How would you rate this resident’s attitude?	65%	30%	5%	3.96 (1.0)
Is this resident a team player?	64%	30%	6%	3.95 (1.0)
How well does this resident demonstrate ethical and professional behavior in the emergency department?	58%	39%	3%	3.88 (0.9)
How would you rate this resident’s interpersonal/communication skills with the ancillary staff (Nurses, techs, clerks)?	62%	28%	10%	3.86 (1.1)
How would you rate this resident’s ability to direct other healthcare workers during resuscitations?*	39%	28%	5%	3.74 (1.0)
How would you rate the clarity of this resident’s orders and discharge instructions?	51%	43%	5%	3.73 (0.9)
How would you rate the resident’s judgment as it applies to patient care (medical decision making)?	52%	42%	6%	3.71 (0.9)
How would you rate this resident’s clinical efficiency and ability to maintain patient flow?	53%	38%	9%	3.69 (1.0)

*SD*, standard deviation.

* 27% of residents were not evaluated on this item.

**Table 2:** Qualitative themes and frequency of each theme in nurses’ assessments of emergency medicine residents.

Themes*	Frequency	Negative	Positive
Communication with nursing	97 (21%)	50	47
Nice and kind	81 (17%)	11	70
Work ethic and efficiency	83 (18%)	52	31
Resident judgment	53 (11%)	26	27
Communication with patients	38 (8%)	17	21
Respect	33 (7%)	19	14
Team player	33 (7%)	8	25
Confidence and leadership	30 (6%)	22	8
Nursing clinical judgment	20 (4%)	8	12

* Text comments often contained multiple themes; therefore, the numbers may add up to greater than 100%.
